# Understanding meal patterns: definitions, methodology and impact on nutrient intake and diet quality

**DOI:** 10.1017/S0954422414000262

**Published:** 2015-06

**Authors:** Rebecca M. Leech, Anthony Worsley, Anna Timperio, Sarah A. McNaughton

**Affiliations:** Centre for Physical Activity and Nutrition Research (C-PAN), School of Exercise and Nutrition Sciences, Deakin University, 221 Burwood Highway, Burwood, VIC3125, Australia

**Keywords:** Meal patterns, Diet quality, Nutrient intake, Diet quality indicators

## Abstract

Traditionally, nutrition research has focused on individual nutrients, and more recently dietary patterns. However, there has been relatively little focus on dietary intake at the level of a ‘meal’. The purpose of the present paper was to review the literature on adults' meal patterns, including how meal patterns have previously been defined and their associations with nutrient intakes and diet quality. For this narrative literature review, a comprehensive search of electronic databases was undertaken to identify studies in adults aged ≥  19 years that have investigated meal patterns and their association with nutrient intakes and/or diet quality. To date, different approaches have been used to define meals with little investigation of how these definitions influence the characterisation of meal patterns. This review identified thirty-four and fourteen studies that have examined associations between adults' meals patterns, nutrient intakes and diet quality, respectively. Most studies defined meals using a participant-identified approach, but varied in the additional criteria used to determine individual meals, snacks and/or eating occasions. Studies also varied in the types of meal patterns, nutrients and diet quality indicators examined. The most consistent finding was an inverse association between skipping breakfast and diet quality. No consistent association was found for other meal patterns, and little research has examined how meal timing is associated with diet quality. In conclusion, an understanding of the influence of different meal definitions on the characterisation of meal patterns will facilitate the interpretation of the existing literature, and may provide guidance on the most appropriate definitions to use.

## Introduction

It is widely recognised that a nutritionally sound diet is fundamental to human health and wellbeing across the lifespan^(^ ^1^ ^)^. A poor diet contributes to poor health and is a well-established, modifiable risk factor for the development of non-communicable diseases, which are leading causes of death globally^(^ ^1^ ^)^. Traditionally, research has focused on the relationship between individual nutrients and health outcomes, yet this approach has often resulted in conflicting findings^(^ ^2^ ^)^. Hence there has been a gradual shift in the past decade towards less reductionist approaches to examining diet–disease relationships (for example, dietary patterns analysis) that better capture the interaction of nutrients and bioactive compounds within the whole diet^(^ ^2^ ^,^ ^3^ ^)^. However, people consume combinations of foods as meals and snacks rather than as individual foods and nutrients. Understanding the nutritional composition of meals and the ways in which different meal patterns make an impact on diet quality might help to elucidate important diet–disease relationships. Moreover, a meals-based approach could complement current dietary advice, which currently uses a food-based framework (for example, the Australian Dietary Guidelines)^(^ ^4^ ^)^ to assist populations in achieving the recommended daily intakes of foods and nutrients. That is, dietary advice in the context of meals could help populations with their daily meal preparation and therefore be a more practical and salient way to assist populations to follow dietary guidelines.

Most of the research in this area, however, has focused on how different meal patterns (also referred to as eating patterns) make an impact on energy balance and weight status^(^ ^5^ ^,^ ^6^ ^)^. An oft-cited drawback to interpreting the evidence from these studies has been the different approaches employed to operationally define meals and/or snacks^(^ ^5^ ^,^ ^7^ ^)^. However, previous reviews of the impact of different definitions on the interpretation of meal pattern studies have examined snacking only^(^ ^7^ ^,^ ^8^ ^)^. Moreover, to date there has been no comprehensive review of studies investigating associations between meal patterns and diet quality; previous reviews have focused on dietary contributions in relation to eating frequency or snacking^(^ ^8^ ^,^ ^9^ ^)^.

Therefore, the primary purpose of the present paper is to provide an overview and critique of meal pattern research, including previous approaches to the characterisation, definition and measurement of ‘meals’. Second, the potential implication of these approaches will be further examined in a critical review of the literature of the contributions of meal patterns to energy and nutrient intakes and overall diet quality among adults.

## Characterisation of meals

The term ‘meal patterns’ is an overarching construct that is often used to describe individuals' eating patterns at the level of a ‘meal’, such as a main meal (for example, breakfast, lunch or dinner) or a smaller-sized meal (for example, supper or snack). The neutral terms ‘eating occasion’ (EO) or ‘eating event’ are also used to describe any occasion where food or drink is ingested, and therefore incorporates all meal types. Meals have been described according to three constructs: (1) patterning (for example, frequency, spacing, regularity, skipping, timing); (2) format (for example, types of food combinations, sequencing of foods, nutrient profile/content); and (3) context (for example, eating with others or with the family, eating in front of the television or out of the home) (Table 1)^(^ ^10^ ^–^ ^13^ ^)^. Table 1 ^(^ ^14^ ^–^ ^34^ ^)^ provides an overview of these constructs, including a description of the different meal patterns variables that have been examined previously along with their corresponding operational definitions. Examples of how meals have been measured in past studies are also presented in Table 1.

**Table 1:** Overview of the three meal pattern constructs, and examples of variables currently assessed in the literature and the assessment methods that have been used to collect the meal pattern data

Construct	Variable	Operational definition(s)	Example(s) of methods
Patterning	Frequency of EO (meals and snacks)	Mean number of EO/meals/snacks per d^(^ ^14^ ^)^	Dietary recall (24 h)^(^ ^14^ ^)^ Weekly food diary^(^ ^15^ ^)^ Meal patterns questionnaire^(^ ^16^ ^)^ Single questionnaire items^(^ ^17^ ^)^
	Spacing of EO	Mean time between EO^(^ ^14^ ^)^	Dietary recall (24 h)^(^ ^14^ ^)^
	Regularity of meals	Consistency of EO frequency and spacing^(^ ^18^ ^)^ Usually eats breakfast, lunch and dinner each day^(^ ^19^ ^–^ ^21^ ^)^	Semi-quantitative food records^(^ ^18^ ^)^ Single questionnaire items^(^ ^19^ ^,^ ^20^ ^)^ Food records (3 d)^(^ ^21^ ^)^
	Meal skipping	Usually omits breakfast, lunch or dinner^(^ ^17^ ^,^ ^22^ ^)^	Single questionnaire items^(^ ^17^ ^)^ Meal patterns questionnaire^(^ ^22^ ^)^ Dietary recall (24 h)^(^ ^23^ ^)^
	Meal timing	The timing of breakfast, lunch or dinner (early/late)^(^ ^24^ ^)^ Time-of-day wherein majority of daily EI is consumed (morning, midday, evening/late)^(^ ^25^ ^–^ ^27^ ^)^ Late-night eating (eating after going to bed)^(^ ^28^ ^)^	Food records (7 d)^(^ ^24^ ^)^ Prescribed diet (intervention studies)^(^ ^26^ ^)^ Dietary recall (24 h)^(^ ^27^ ^)^ Single questionnaire items^(^ ^28^ ^)^
Format	Meal food type/combinations	Classifications of combinations of foods in meals^(^ ^29^ ^)^	Food records (7 d)^(^ ^29^ ^)^
	Meal food sequencing	Temporal distribution of consumption of food groups and intake of energy and nutrients within a meal^(^ ^30^ ^)^	Food records (2 d)^(^ ^30^ ^)^
	Nutrient composition	Energy, protein, fat and carbohydrate composition of a meal^(^ ^25^ ^,^ ^26^ ^,^ ^31^ ^)^	Prescribed diet (intervention studies)^(^ ^26^ ^)^ Weekly food diary^(^ ^31^ ^)^
Context	Presence of others at a meal (for example, friends/family)	Types of food eaten in different social contexts (for example, alone *v.* with others)^(^ ^32^ ^)^ Macronutrient composition of meals eaten alone *v.* with others^(^ ^33^ ^)^	Personal digital assistants^(^ ^32^ ^)^ Weekly food diary^(^ ^33^ ^)^
	Eating while doing activities (for example, watching TV)	Types of food consumed while watching TV *v.* other activities^(^ ^32^ ^,^ ^34^ ^)^	Personal digital assistants^(^ ^32^ ^)^ Food records and ecological momentary assessment^(^ ^34^ ^)^
	Meal location (for example, eating at home *v.* eating out)	Energy and macronutrient content of meals consumed at home *v.* out of the home^(^ ^30^ ^)^ Types of food consumed by location^(^ ^32^ ^)^	Food records (2 d)^(^ ^30^ ^)^ Personal digital assistants^(^ ^32^ ^)^

EO, eating occasions; EI, energy intake; TV, television.

## Meal definitions

To date, a variety of approaches has been used in the literature to define EO (meals and snacks). The approaches are summarised below and in Table 2 ^(^ ^11^ ^,^ ^16^ ^,^ ^34^ ^–^ ^42^ ^)^. The main approaches to defining meals are: participant-identified, time-of-day, food-based classification (FBC) and neutral. These definitions, along with examples from the literature and their respective advantages and disadvantages, are discussed below.

**Table 2:** Summary of different approaches used to define different eating occasions (EO)

Approach	Description	Examples in the literature	Advantages	Disadvantages
Time-of-day	According to time intervals during which EO occur (for example, morning, mid-morning, midday, mid-afternoon, evening and late evening)	Participants record all foods and drinks consumed according to six time slots/feeding periods^(^ ^35^ ^,^ ^36^ ^)^	Easy to apply and understand. Emphasis on ‘when’ foods are eaten	Bias towards traditional eating patterns. Does not cater for individuals with varied meal times (for example, shift workers)
Participant-identified	Participants report their own EO, usually from a list of pre-specified meal labels	Participants identify EO as a main meal, light meal, snack or drink only^(^ ^16^ ^)^Participants identify EO from the following labels: breakfast, lunch, dinner/supper or snack^(^ ^37^ ^)^	Avoids use of a complex ‘*a priori*’ definition of different EO	Definition not standardised. Meal labels may be influenced by the researcher's existing understanding of eating patterns and introduce researcher bias
Food-based classification	Foods are firstly categorised into food categories based on their nutritional profile. Food category combinations determine the type of EO	Six EO types ranging from a complete meal (high nutrient density; contains both animal and plant foods) to a low-quality snack (low nutrient density)^(^ ^38^ ^)^. Meals and snacks defined by ‘core’ and ‘non-core’ foods, respectively^(^ ^39^ ^)^	Provides information on the nutritional quality as well as the patterning of eating events	Complex categorisation system. Differences in classification criteria of meals and snacks may limit comparison across studies
Neutral	A neutral term (for example, ‘eating event’) is used to collect meal pattern data. Aspects of meal patterns are then analysed using standardised criteria	An EO is any occasion when food (excluding drinks only)^(^ ^11^ ^)^ or food/drink is consumed^(^ ^34^ ^)^An EO is considered separate if it occurs>15 min apart^(^ ^40^ ^,^ ^41^ ^)^An EO is an event that provides a minimum of 50 kcal (210 kJ) and is separated by 15 min^(^ ^42^ ^)^	Standardised and consistent. Findings may be more comparable over time and across studies	Loss of qualitative information about individuals' perceptions of what constitutes a meal/snack. Differences in ‘standardised criteria’ may limit comparison across studies

### Time-of-day

As the name implies, this approach defines meals according to the time-of-day in which food was consumed. Explicitly, a ‘meal’ may be defined as the largest EO occurring between 06.00–10.00, 12.00–15.00 and 18.00–21.00 hours, with smaller EO and EO falling outside of these times considered as snacks^(^ ^43^ ^)^. While this approach is easy to understand and apply, the time parameters used are not always explicit, the number of meals per d is usually restricted to a maximum of three and it does not capture meals eaten at unusual times, such as among shift workers^(^ ^35^ ^,^ ^36^ ^)^. Ultimately, a time-of-day definition requires a measure of time of eating. It is also subject to bias of the researcher, as the time intervals to define a meal or snack are often based on their understanding of eating patterns, potentially influenced by local or cultural factors^(^ ^44^ ^)^

### Participant-identified

This definition relies on the respondent to identify an EO as a meal or snack, often from a list of pre-specified meal labels (for example, breakfast, lunch, dinner/supper or snack)^(^ ^44^ ^)^. While this definition avoids the imposition of a complex criterion to classify EO as meals or snacks^(^ ^45^ ^–^ ^47^ ^)^, it is not standardised due to subjectivity in participants' allocation of an eating event as a meal or snack^(^ ^44^ ^,^ ^48^ ^)^. Chamontin *et al.* ^(^ ^49^ ^)^ showed that the word ‘snack’, when used in its verb form (for example, ‘When did you last snack?’), elicited different conceptual responses from participants than when snack was used as a noun (for example, ‘When did you last have a snack?’). However, not all studies ask respondents to identify the EO^(^ ^43^ ^)^.

### Food-based classification

Lennernäs & Andersson^(^ ^38^ ^)^ developed the concept of a FBC of EO intended to reflect both qualitative and quantitative aspects of meal patterns. Initially, foods consumed were sorted into seven food categories that differed by nutritional profile (for example, animal/plant origin, nutrient density, energy density) and second, depending on the combination of food categories consumed, eating events were classified as one of six types of EO ranging from a ‘complete meal’ to a ‘low-quality snack’. Another variation of the FBC system, based on ‘core’ and ‘non-core’ foods has since been developed^(^ ^39^ ^)^, but generally the FBC of EO has had limited uptake, probably due to the complexity of the FBC criteria. While this definition of a meal can capture the types of foods eaten, the researcher must decide which criteria should be used to classify meals and snacks (for example, a criterion based on different nutrient profiles *v.* a criterion based on the energy density of foods).

### Neutral

In 1999, Mäkelä *et al.* ^(^ ^11^ ^)^ recognised that conventional meal labels are culturally laden and therefore may mean different things for people from different cultural backgrounds. This led the authors to use the neutral term ‘eating event’ for an occasion where food was consumed. Once empirical data had been collected, different dimensions of meal patterns using standardised criteria (for example, time-of-day, number of hot/cold eating events) were used to describe the data. The advantage of a neutral definition is that it can be standardised and can allow for comparison across different population groups and cultures. However, despite this neutral definition, additional criteria have been applied in the literature with regards to the time intervals between EO, a minimum-energy criterion to define each individual EO and whether beverage-only EO are included or excluded. It is important to note that these additional criteria have also been applied to the time-of-day and participant-identified definitions in order to define an ‘individual’ or ‘separate’ meal and/or snack^(^ ^15^ ^,^ ^50^ ^,^ ^51^ ^)^, thus adding another layer of diversity to these meal definitions.

When the time of eating is recorded, researchers must decide how to delineate separate EO^(^ ^35^ ^,^ ^52^ ^)^. Indeed, the period of time used to separate different EO varies across studies, with intervals of 15 min^(^ ^40^ ^,^ ^42^ ^,^ ^50^ ^)^, 30 min^(^ ^53^ ^)^ or 45 min^(^ ^15^ ^)^ or 1 h^(^ ^51^ ^)^ reported. Some studies also include a minimum energy criterion as part of the meal definition. For example, in some studies, EO were only treated as an EO if they contributed a minimum amount of energy (for example, 210 kJ)^(^ ^15^ ^,^ ^42^ ^,^ ^54^ ^)^. These variations in criteria are likely to make an impact on the frequency, spacing and nutritional contribution of the EO reported and on associations with health outcomes. In support of this, Murakami & Livingstone^(^ ^55^ ^)^ found the number of reported EO per d was reduced by two or more EO for both men and women after applying a minimum-energy criterion of 210 kJ. In the same study, the different definitions of an EO greatly affected the results of the association between eating frequency and BMI and waist circumference.

The methodological differences in spacing between EO and energy content may also indirectly influence the inclusion^(^ ^50^ ^)^ or exclusion^(^ ^56^ ^)^ of beverage-only occasions as part of the meal definition. A larger time interval criterion applied to 24 h recall data to separate individual EO may not be able to capture smaller EO (including beverage-only occasions). The findings from one study suggest that smaller intervals may also be useful to detect important changes in energy intake (EI) from beverage-only occasions over time^(^ ^14^ ^)^.

## Measurement of meals

There have been a number of different approaches to the measurement of meals. Much data on meal patterns have been derived from dietary assessment methods such as 24 h recalls and food records. These methods provide detailed information on the types and quantities of food/beverages consumed and, usually, time of consumption^(^ ^14^ ^,^ ^15^ ^,^ ^36^ ^,^ ^50^ ^,^ ^57^ ^)^.

During a 24 h recall, participants may be asked to report the type of EO as a main meal or a snack^(^ ^14^ ^,^ ^50^ ^,^ ^57^ ^)^, whereas food records are often segregated by the researcher into meal time slots (for example, pre-breakfast, breakfast, mid-morning, etc.)^(^ ^35^ ^,^ ^36^ ^)^. Contextual information is not always collected as part of the recall or food record and thus only examination of meal patterning may be possible. While meal format could be examined with this type of data, little research in this area has been conducted. One possible reason for this is that there has been little exploration of statistical techniques that are able to examine complex combinations and sequencing of foods at a meal. Hearty & Gibney^(^ ^29^ ^)^ explored the potential use of supervised data-mining techniques in meal pattern analysis, specifically to predict diet quality based on different combinations of foods at a meal. However, to our knowledge, this is the only study that has applied these analytic tools in meal pattern analysis.

Many food diaries collect data on time of eating, and/or self-identified meals, and similar to 24 h recalls, some collect contextual information (for example, location of eating, presence of others). The weekly food diary method developed by de Castro^(^ ^58^ ^)^, in addition to the time and amount of food eaten over a 7 d period, asks participants to record detailed contextual information (for example, mood and hunger levels before eating, the number and nature of other people eating with them). While this method elicits rich contextual information, participant burden is high, thus reducing its practicality in larger-scale studies. In a recent study, participants used personal digital assistant devices to record real-time information on dietary intake, EO type, location and context^(^ ^32^ ^)^. As a result, the researchers were able to assess contextual influences on the types of foods that participants consumed at an EO. However, while this type of assessment method lends itself to the examination of meal patterning, format and context, these ‘real-time’ assessment devices have not yet been extensively developed^(^ ^59^ ^)^.

FFQ are also commonly used to collect dietary data, particularly in large-scale studies^(^ ^60^ ^)^. While FFQ provide estimates of the frequency and types of foods that are usually consumed, they do not provide data that directly allow examination of EO, and additional questionnaires^(^ ^16^ ^,^ ^56^ ^)^ or short questionnaire items^(^ ^17^ ^)^ have been used to collect information on meal patterns. Example of questionnaire items include: ‘Indicate the times of day you usually eat’^(^ ^17^ ^)^, ‘Do you eat regular breakfast, lunch and dinner or evening meal each day?’^(^ ^19^ ^)^ or ‘Do you usually have the following meals (breakfast, lunch, snack, dinner, evening snack)?’^(^ ^20^ ^)^. Some questionnaires may only ask about ‘eating’ frequency, and thus may not capture beverage-only EO. The reliability and validity of meal pattern questionnaires are often not reported^(^ ^16^ ^)^

## Associations between meal patterns, nutrient intakes and overall diet quality

Due to the current limited methods available to collect meal pattern data, most research to date has examined meal patterning^(^ ^5^ ^,^ ^61^ ^)^, with relatively little focus on meal format^(^ ^29^ ^)^ and context^(^ ^32^ ^)^. As stated previously, studies on meal patterning have examined meal frequency, spacing, skipping and timing. However, these studies have differed in their approach to defining meals, and even within a given approach, there have been differences in the delineation of individual EO, meals and/or snacks. The ways these different methodological differences affect the characterisation of meal patterns have been little explored^(^ ^55^ ^)^ and, to the best of our knowledge, how these differences affect the associations between meal patterns and nutrient intakes or diet quality has not previously been examined. Understanding the relationships between adults' meal patterns and nutrient intakes and diet quality is necessary to determine if they are markers of the healthiness and variety of the whole diet^(^ ^62^ ^)^. Therefore, the associations between ‘meal patterning’, nutrient intake and overall diet quality among adults were examined considering the impact of different meal definitions used for the characterisation of meal patterns.

A literature search was undertaken in the PubMed and EMBASE electronic databases using the following terms: meal, snack, breakfast, lunch, dinner, eating frequency, eating pattern, eating behavior, eating behaviour, eating occasion, eating episode, diet quality, dietary quality, dietary pattern, dietary behavior, dietary behaviour, nutritional quality, dietary intake, food intake, energy intake, nutrient, macronutrient, dietary composition and nutritional composition. The search terms were limited to the title/abstract and the following filters were applied: journal article, humans, adult and English. Two searches were conducted; the first between February and May 2013 and the second between February and April 2014. The criteria for inclusion in the review were: original research studies that examined the nutritional contributions of meal patterns or associations between meal patterns and nutrient intakes and/or overall diet quality in free-living, healthy men and women aged 19 years and over. Diet quality was defined as the quality of a individual's overall food intake determined by compliance with national dietary guidelines or an *a priori* diet quality score^(^ ^63^ ^)^. Studies that examined populations with conditions or circumstances that may affect meal patterns (for example, elite athletes, shift-workers, individuals with chronic diseases, recipients of meal programmes and pregnant or breast-feeding women), or examined associations with EI only, were excluded.

### Characteristics of studies which examined associations between meal patterns and nutrient intakes

A total of thirty-four studies (Table 3) were identified which examined the nutritional contribution of different meal patterns, in adults. However, only thirteen of these examined more than one micronutrient^(^ ^23^ ^,^ ^30^ ^,^ ^46^ ^,^ ^47^ ^,^ ^52^ ^,^ ^64^ ^–^ ^71^ ^)^. All except two studies^(^ ^35^ ^,^ ^72^ ^)^ were cross-sectional. Of the studies, fifteen and five studies were conducted in the USA^(^ ^41^ ^,^ ^46^ ^,^ ^48^ ^,^ ^52^ ^,^ ^57^ ^,^ ^68^ ^,^ ^70^ ^,^ ^72^ ^–^ ^79^ ^)^ and Scandinavia^(^ ^45^ ^,^ ^47^ ^,^ ^53^ ^,^ ^65^ ^,^ ^80^ ^)^, respectively, with fewer studies conducted in Western Europe^(^ ^15^ ^,^ ^30^ ^,^ ^67^ ^,^ ^81^ ^)^, the UK^(^ ^35^ ^,^ ^40^ ^,^ ^82^ ^)^, East Asia^(^ ^64^ ^,^ ^71^ ^,^ ^83^ ^)^, Australia^(^ ^36^ ^,^ ^69^ ^)^, Canada^(^ ^66^ ^)^ and Brazil^(^ ^84^ ^)^. Meal patterns were mostly participant-identified^(^ ^15^ ^,^ ^23^ ^,^ ^45^ ^–^ ^48^ ^,^ ^52^ ^,^ ^53^ ^,^ ^57^ ^,^ ^66^ ^,^ ^69^ ^,^ ^70^ ^,^ ^75^ ^,^ ^77^ ^,^ ^78^ ^,^ ^82^ ^)^, although these studies varied in the additional criteria used to determine an individual EO, meal and/or snack, and their treatment of beverages. For example, eleven studies^(^ ^23^ ^,^ ^45^ ^–^ ^47^ ^,^ ^52^ ^,^ ^66^ ^,^ ^69^ ^,^ ^75^ ^,^ ^78^ ^,^ ^80^ ^,^ ^82^ ^)^ applied no additional criteria, whereas in other studies, EO were delineated using 15-min time intervals^(^ ^57^ ^,^ ^70^ ^,^ ^77^ ^)^, a 30-min interval^(^ ^53^ ^)^, a 45-min interval plus a 50 kcal (210 kJ) energy criterion^(^ ^15^ ^)^ and a 59-min interval (applied to meals only)^(^ ^48^ ^)^. All beverage types (energy and non-energy) could constitute an individual EO in nine studies^(^ ^35^ ^,^ ^45^ ^–^ ^47^ ^,^ ^53^ ^,^ ^66^ ^,^ ^70^ ^,^ ^75^ ^,^ ^77^ ^)^, whereas other studies excluded water beverages^(^ ^23^ ^,^ ^52^ ^)^, non-nutritive beverages (for example, water, tea, black coffee)^(^ ^80^ ^)^ or did not address/include beverages as part of the definition^(^ ^48^ ^,^ ^57^ ^,^ ^71^ ^,^ ^78^ ^,^ ^82^ ^)^. Time-of-day definitions were also common^(^ ^35^ ^,^ ^36^ ^,^ ^64^ ^,^ ^67^ ^,^ ^72^ ^,^ ^76^ ^)^ as well as a combination of two definitions (for example, self-identified and time-of-day, or time-of-day and type/combination of foods eaten)^(^ ^30^ ^,^ ^65^ ^,^ ^68^ ^,^ ^73^ ^,^ ^81^ ^)^.

**Table 3:** Summary of studies that have examined the contribution of meal patterns to macronutrient and/or other nutrient intakes

First author (year)	Country and study design	Sample	Aspect(s) of meal patterns examined	Diet and meal pattern measure	Meal or snack definition	Macronutrients	Other dietary components	Covariates	Selected key findings
Almoosawi (2012)^(^ ^35^ ^)^	UK Prospective (17 years)	562 men and 691 women, 36 years at baseline in 1982	Distribution of nutrient intake from EO across the day	5 d FR	Time-of-day*	Protein, fat, CHO	EI, NSP, alcohol	Stratified by sex	The lunch and evening meal contributed the greatest proportion of daily EI and nutrient intake compared with other EO. Between 1982 and 1999 there was a shift towards greater EI, macronutrient intake in the mid-afternoon and evening
Barr (2012)^(^ ^66^ ^)^	Canada C/S	8973 men and 10 940 women, ≥ 19 years	Breakfast skipping	24HR	Participant-identified*	Protein, fat, CHO, sugars, SFA, MUFA, PUFA	EI, fibre, cholesterol, vitamins A, B_6_, B_12_, C and D, thiamin, riboflavin, niacin, folate, Zn, Ca, Fe, Mg, P, Na and K	EI, age, sex, race, education, PA, food security, language spoken at home, smoking and supplement use	Breakfast skippers had significantly lower intakes for energy, niacin, folate and vitamin C, fibre, thiamin, riboflavin, Fe, Mg, P and K than breakfast consumers. Breakfast skippers had a significantly higher prevalence of inadequate total intakes of vitamin D, Ca, vitamin A and Mg than breakfast consumers
Basdevant (1993)^(^ ^67^ ^)^	France	273 obese women, ≥ 18 years (mean age 41 (se 12) years)	Snacking ( ≥ 15 % EI from snacks) *v.* non-snacking	Diet history interview	Time-of-day	Protein, fat, CHO	EI	–	Snackers had significantly higher total daily EI and EI from meals than non-snackers (*P*< 0·05). Snackers also consumed a greater percentage of energy as fat but less energy as protein than non-snackers (*P*< 0·05)
Bellisle (2003)^(^ ^15^ ^)^	France C/S	15 men and 39 women, 26–58 years	Meals and snack frequency. Distribution of nutrient intake from meals and snacks across the day	4 × 7 d FR across four seasons	Participant-identified*	Protein, fat, CHO	EI, alcohol	–	Snacks contributed a significantly greater percentage of CHO but less fat and protein than meals. EI and macronutrient intake highest during 10.00–14.00 hours and 18.00–20.00 hours
Berner (2013)^(^ ^73^ ^)^	USA C/S	893 men and 875 women, ≥ 51 years	Distribution of protein intake from meals and snacks across the day	2 × 24HR	Self-identified and time-of-day*	Protein, animal protein		Stratified by age group and sex	Percentage of protein intake was highest at dinner (about 44–48 %) and lowest at snacks (about 10–12 %). Percentage of protein from animal sources was also highest at dinner (about 65–68 %) and lowest at snacks (about 29–32 %)
Bertéus Forslund (2005)^(^ ^45^ ^)^	Sweden C/S	Obese group: 1891 men and 2368 women, 30–60 years; reference group: 505 men and 587 women, 37–60 years	Meal, snack and total EO frequency*	FFQ and meal pattern Q	Participant-identified*	Protein, fat, CHO	EI, fibre, alcohol	Age, PA and stratified by sex	EI increased with increasing number of meals in obese men and women only and snacking frequency was associated with higher EI in obese and reference men and women. The proportion of EI from protein decreased while the proportion from fat increased by increasing snacking category among obese men and women
Coates (2002)^(^ ^72^ ^)^	USA Case–control	2380 healthy control men and women, 30–79 years	Eating frequency	Dietary history interview	Time-of-day		EI, Ca, fibre		Control men and women eating 1–2 times/d were more likely to have lower intakes of energy, Ca and fibre than those eating ≥ 3 times/d (*P*< 0·001)
Dattilo (2011)^(^ ^84^ ^)^	Brazil	24 men and 28 women, 19–45 years	Meal distribution across the day/meal timing	Dietary recall (recall period not given)	Not clear	Protein, fat, CHO	EI	Stratified by sex	Among women only, EI was significantly higher (*P*< 0·01) at lunch than at breakfast, snacks and at supper. CHO and protein intake at night (among men) and afternoon (among women) was higher in than in the morning. A higher proportion of daily EI in the afternoon and evening was associated with lower and higher overall EI, respectively (*P*< 0·05)
de Castro (2004)^(^ ^79^ ^)^	USA C/S	375 men and 492 women, mean age 36.3 (sd 13·8) years	Meal distribution across the day/meal timing	7 d FR	Neutral*	Protein, fat, CHO	EI	Stratified by sex. Sensitivity analysis excluding energy under-reporters	Intakes per meal of energy as CHO, fat, protein and alcohol were significantly higher in the evening period (18.00–22.00 hours) than the other four periods of the day (*P*< 0·05). Among both sexes, a higher proportion of daily EI in the morning and evening was associated with lower and higher overall EI, respectively (*P*< 0·01)
Deshmukh-Taskar (2010)^(^ ^23^ ^)^	USA C/S	2615 men and women, 20–39 years	Breakfast skipping	24HR	Participant-identified*	Protein, fat, CHO, added and total sugars, SFA, MUFA, PUFA, discretionary oils and solid fats	EI, fibre, vitamins A, B_6_, B_12_, C, D and E, thiamin, riboflavin, niacin, folate, Zn, Ca, Fe, Mg, P, Na, K and cholesterol	EI, age, ethnicity, sex, sex × ethnicity, poverty income ratio, smoking, PA, marital status and alcohol intake	Compared with breakfast consumers, total energy, dietary fibre, vitamin A, thiamin, riboflavin, vitamin B_12_, folate, Ca, P, Mg and K intakes were significantly lower in breakfast skippers (*P*< 0·0001). Nutrient adequacy ratios for vitamins A and C, Ca, Mg, K and fibre were also significantly lower among breakfast skippers (*P*< 0·01)
Drummond (1998)^(^ ^40^ ^)^	Scotland C/S	48 men and 47 women, 20–55 years	Eating frequency	7 d FR†	Neutral‡	Protein, CHO, fat, sugar	EI, alcohol	Stratified by sex	Eating frequency was positively correlated with total EI (*r* 0·31, *P*= 0·01) among women only and percentage of energy from CHO (women: *r* 0·38, *P*= 0·02 and men: *r* 0·3, *P*= 0·05)
Duval (2008)^(^ ^41^ ^)^	USA C/S	85 women, 47–56 years	Eating frequency	7 d FR†	Neutral	Protein, CHO, fat	EI, alcohol		Eating frequency was positively correlated with total EI (*r* 0·41, *P*= 0·005) and percentage of energy from CHO (*r* 0·21, *P*= 0·045) and total weight of CHO (*r* 0·37, *P*= 0·001) and protein (*r* 0·31, *P*= 0·005)
Edelstein (1992)^(^ ^78^ ^)^	USA C/S	2034 white adults, 50–89 years	Eating frequency	FFQ Meal patterns: one Q item	Participant-defined	Fat, SFA	EI, cholesterol, fibre	Age and sex	Eating frequency was positively associated with EI, total fat and SFA (*P*< 0·001) and total fibre (*P*= 0·012)
Hampl (2003)^(^ ^46^ ^)^	USA C/S	1756 men and 1511 women, 39–43 years	Snacking frequency and timing	2 × 24HR	Participant-identified*	CHO, fat, protein	EI, all nutrients with an RDA except vitamins D and K, Se and I	EI stratified by sex and ‘snacker’ type	Male and female multiple snackers had lower intakes of protein, cholesterol and Na, but higher EI and Ca intake compared with non-snackers. Female evening snackers had significantly higher EI than did morning snackers
Holmbäck (2010)^(^ ^80^ ^)^	Sweden C/S	1355 men and 1654 women, 47–68 years	Eating frequency	Diet history interview† Meal patterns: Q	Participant-identified*	CHO, fat, protein	EI, fibre, Fe, Ca, Mg, β-carotene, ascorbic acid, vitamin E, folate and alcohol	Stratified by sex and excluded individuals with past food habit change	Total EI and percentage energy from CHO significantly increased with increased eating frequency (*P*< 0·01). Percentage energy from fat (women only), protein and fibre density decreased. Nutrient densities of vitamin C, folate and Fe were significantly higher among women who ate ≥ 6 times/d (*P*< 0·01)
Howarth (2007)^(^ ^48^ ^)^	USA C/S	1792 young adults, 20–59 years; and 893 older adults, 60–90 years	Meal and snack frequency, meal skipping	2 × 24HR†	Participant-identified	CHO, fat, protein	EI, fibre, fibre density	Age group	Among both age groups lunch and dinner provided the highest proportion of EI from protein and fat and snacks provided the least amount of fibre density Dinner provided the highest fibre density among younger adults. Among older adults, breakfast was higher in fibre density
Kant (1997)^(^ ^76^ ^)^	USA Prospective (mean follow-up 10.1 years)	2580 men and 4567 women, 25–74 years	Meal timing (evening eating)	24HR	Time-of-day (eating after 17.00 hours)	CHO, fat, protein	EI, alcohol	Stratified by sex, age, and EI (unless EI was the dependent variable)	C/S analysis of baseline data showed that with increasing percentage of energy consumed after 17.00 hours, mean daily energy and alcohol intake increased and percentage energy from CHO decreased
Kearney (2001)^(^ ^30^ ^)^	Holland C/S	About 6000 participants (age/sex not provided)	Contribution of meals *v.* snacks to nutrient intakes	2 d FR	Time-of-day and type of foods eaten (for example, lunch = bread meal; dinner = hot meal)	Protein, animal and vegetable protein, fat, SFA, MUFA, PUFA, CHO	EI, cholesterol, fibre, Ca, P, Fe, haem Fe, non-haem Fe, Zn and vitamins B_1_, B_2_, B_6_, D, E and C	–	The (hot) dinner meal was the main contributor to the intake of all micronutrients except Ca. The dinner meal also provided 71 % of haem Fe and the lunch (bread) meal was the main contributor of Ca intakes
Kerver (2006)^(^ ^52^ ^)^	USA C/S	15 978 adults, ≥ 20 years	Eating frequency, meal skipping	24HR	Participant-identified*	Protein, CHO, fat	EI, cholesterol, vitamins B_6_ and C, folic acid, Fe, Ca, Mg, Na, K, fibre	Age group, sex, ethnicity, income, smoking status, alcohol intake, vitamin and mineral supplement use, BMI, PA	More frequent eaters had higher intakes of CHO, folic acid, vitamin C, Ca, Mg, Fe, K and fibre and lower intakes of fat, protein and cholesterol than those who ate 1–2 times/d Breakfast skippers had the lowest intake of all micronutrients except Na
Khan (1982)^(^ ^75^ ^)^	USA C/S	71 men and 179 women students, ≤ 25 years	Contribution of meals *v.* snacks to energy and nutrient intakes	Q (based on a 24HR)	Participant-identified*	Protein	EI, Ca, Fe, vitamins A and C, thiamin, riboflavin, niacin	Stratified by sex	Snacks contributed significantly to the percentage of the RDA for protein (13·4–24·1 %), Ca (9·9–20·2 %), Fe (11·3–34·8 %), vitamin C (13·5–29 %), thiamin (12–18 %), riboflavin (12·4–24·7 %) and niacin (14·2–30 %). The contribution of snacks to women's Fe intakes was important as meals only provided about 57·5 % of the RDA
Kim (2010)^(^ ^83^ ^)^	Korea C/S	292 men and 391 women, 20–65 years	Meal and snack frequency. Combinations of meals and snacks	24HR	Participant-identified and time-of-day*	Protein, fat, CHO	EI	Stratified by sex	Absolute energy and CHO intake highest in the three meals plus three snacks group. There were no differences in protein or fat intakes between more frequent snackers *v.* less frequent snackers
Kuroda (2013)^(^ ^71^ ^)^	Japan C/S	275 women students, 19–25 years	Meal skipping	Diet: diet history Q Meal patterns: Q	Participant-identified	Protein, fat, CHO	EI, Ca, phosphate, vitamins D and K	–	Skipping any meal was negatively correlated with total EI (*P*< 0·05). Skipping breakfast was negatively correlated with the absolute intake of all nutrients examined (*P*< 0·05). Skipping lunch or supper was negatively correlated (*P*< 0·05) with absolute CHO intake and vitamin K (lunch only) and vitamin D intake (supper only)
Min (2011)^(^ ^64^ ^)^	Korea C/S	118 men and 297 women, 30–50 years	Breakfast skipping	1 × 24HR and 2 d FR (also included a weekend day)	Time-of-day*	CHO, protein, fat	Cholesterol, fibre, Ca, P, Fe, Na, K, Zn, folate, vitamins A, C, E, B_1_, B_2_ and B_3_	Age, sex and EI	Those who skipped breakfast on two or more of the days (rare breakfast eaters) had lower total EI, fibre, Ca, CHO and K, but higher fat and Fe intakes. Prevalence of not meeting the EAR for Ca, vitamin C and folate was significantly higher among rare breakfast eaters, compared with regular breakfast eaters
Nicklas (1998)^(^ ^68^ ^)^	USA C/S	504 men and women, 19–28 years	Breakfast skipping	24HR	Time-of-day and type of food(s) consumed	CHO, sugars, sucrose, lactose, fructose, protein and vegetable protein, fat, SFA, PUFA, MUFA	Fibre, starch, vitamins A, B_6_, B_12_, C and D, niacin, thiamin, folacin, Ca, riboflavin and P		Breakfast skippers had significantly lower total daily EI (*P*< 0·0001) and significantly lower protein (*P*< 0·05), SFA (*P*< 0·01) and lactose per 1000 kcal (4184 kJ) than breakfast consumers (*P*< 0·0001). A significantly higher percentage of breakfast consumers did not meet two-thirds of the RDA for all vitamins and minerals that were examined
Ovaskainen (2006)^(^ ^47^ ^)^	Finland C/S	912 men and 1095 women, 25–64 years	Meal and snack frequency	48 h recall	Participant-identified*	Fat, protein, CHO, sugars, sucrose	EI, vitamins A, C, E and D, fibre, Ca, Fe, Mg, K, Na, alcohol	Age group, region. Stratified by sex	A snack-predominant meal pattern was associated with higher energy-adjusted sugar, sucrose and alcohol intakes and lower protein, vitamin E, Fe, K and Na intakes among both men and women (*P*< 0·01)
Ovaskainen (2010)^(^ ^53^ ^)^	Finland Repeat C/S (2002 and 2007)	912 men and 846 women in 2002, 728 men and 1095 women in 2007, 25–64 years	Contribution of meals and snacks to nutrient intake	48 h dietary interview	Participant-identified*	Fat, SFA, sucrose	EI, fibre, vitamins C and E, energy density, energy from alcohol	Age and region. Stratified by sex	There were 5-year increases in the contributions of snacks to vitamin C and fibre intakes (per unit of energy) among men and fibre only among women. For both sexes, 5-year decreases in contributions of percentage EI from SFA and vitamin E were observed for both meals and snacks
Roos (1997)^(^ ^65^ ^)^	Finland C/S	870 men and 991 women, 25–64 years	Adherence to a conventional meal pattern (breakfast, warm lunch, dinner)	3 d FR and Q	Participant-identified and time-of-day and foods eaten warm/cold*	Fat, SFA, CHO, sugar, protein	EI, alcohol, fibre, vitamin C, carotenoids, cholesterol	Age and region. Stratified by sex	Per unit of energy, meals contributed higher intakes of fat, protein, fibre carotenoids and cholesterol but lower intakes of sugar, vitamin C and alcohol than snacks (*P*< 0·05)
Summerbell (1995)^(^ ^36^ ^)^	Australia C/S	71 men and 149 women, 17–60, 39–59 and 65–91 years	Contribution of meals and snacks to nutrient intake	7 d FR	Time-of-day*	Protein, fat, CHO, total sugars	EI, alcohol	Analysed separately by age group	Snacks had a lower EI contribution from protein and fat but a higher EI contribution from total sugars than did meals, consistent across all age groups
Titan (2001)^(^ ^82^ ^)^	England C/S	6890 men and 7776 women, 45–75 years	Eating frequency	FFQ Meal patterns: Q (one item)	Participant-identified	Fat, SFA, MUFA, PUFA, CHO, protein	EI, alcohol	Analysed separately by sex	Eating frequency was associated with higher daily EI and absolute intakes of fat, fatty acids, CHO and protein
Williams (2005)^(^ ^69^ ^)^	Australia C/S	5081 men and 5770 women, ≥ 19 years	Breakfast skipping	24HR Meal patterns: Q (one item)	Participant-identified	Fat, CHO, sugar, protein	EI, fibre cholesterol, vitamins A, C, and E, thiamin, riboflavin, niacin, folate, Zn, Ca, Fe, K and P	Stratified by sex	Compared with breakfast skippers, those who ate breakfast regularly ( ≥ 5 times/week) had higher mean daily intakes for all nutrients and minerals examined except for fat (*P*< 0·05). In older adult ( ≥ 65 years) breakfast skippers, the prevalence of not meeting 70 % of the RDI for almost all nutrients was twice that of regular breakfast eaters
Winkler (1999)^(^ ^81^ ^)^	Germany C/S	899 men, 45–64 years	Eating frequency distribution of nutrition intake across EO	7 d FR	Participant-identified and time-of-day*	Protein, fat, CHO	EI, fibre, Ca, alcohol	–	On 85.2 % of reported days, dinner provided most of the energy. Macronutrient intake was contributed mostly by meals and alcohol was mostly drunk at dinner. Snacks in the afternoon or late in the day contained less protein and fibre than the morning snack
Zizza (2001)^(^ ^57^ ^)^	USA C/S (survey years: 1977–1978, 1989–1991 and 1994–1995)	3789 men and 4706 women, 19–29 years	Snacking (*v.* non-snacking)	1997–1991: 1 × 24HR and 2 d FR; 1994–1995: 2 × 24HR	Participant-identified*	Protein, fat, CHO, SFA	EI	–	Within each survey, compared with non-snackers, snackers had significantly higher intakes of CHO, fat and SFA (*P*< 0·01). In the 1994–1996 survey, snackers had significantly greater protein intakes than non-snackers (*P*< 0·01). However, there were no significant differences when all the above macronutrients were expressed as percentage of EI
Zizza (2007)^(^ ^77^ ^)^	USA C/S	2002 men and women, ≥ 65 years	Snacking (*v.* non snacking)	24HR	Participant-identified*	Protein, CHO, fat, SFA	EI, alcohol	Age, poverty income ratio, sex, race, education, marital status, smoking	Snackers had significantly higher intakes of energy, protein, CHO, fat and SFA, compared with non-snackers (*P*< 0·05). Snacking contributed approximately 25 % of daily energy and CHO intakes, and 20 and 12 % of daily fat and protein intakes, respectively
Zizza (2010)^(^ ^70^ ^)^	USA C/S	2056 men and women, ≥ 65 years	Snack frequency	2 × 24HR	Participant-identified*		Vitamins A, B_6_, B_12_, C, E and K, folate, niacin, β-carotene, Cu, lycopene, Fe, Ca, Zn, P, K, Se	EI, sex, race, education, income, BMI	With increasing snack frequency, mean daily intakes of vitamins A, C and E, β-carotene, Mg, Cu and K significantly increased, whereas Se intakes significantly decreased (*P*< 0·05)

EO, eating occasion; FR, food record; CHO, carbohydrate; EI, energy intake; C/S, cross-sectional; 24HR, 24 h recall; PA, physical activity;Q, questionnaire; RDA, recommended daily allowance; EAR, estimated average requirement; RDI, recommended daily intake.

* Beverages could qualify as a separate eating occasion.

† Energy misreporters or under-reporters excluded from analyses.

‡ Milk in excess of 0·5 pints (284 ml) was the only beverage that could qualify as a separate eating occasion.

The most common methods used to assess both dietary intake and meal patterns were 24 h recalls^(^ ^23^ ^,^ ^46^ ^,^ ^48^ ^,^ ^52^ ^,^ ^57^ ^,^ ^66^ ^,^ ^68^ ^–^ ^70^ ^,^ ^73^ ^,^ ^76^ ^,^ ^77^ ^,^ ^83^ ^)^ or food records (2–7 d)^(^ ^15^ ^,^ ^30^ ^,^ ^35^ ^,^ ^36^ ^,^ ^40^ ^,^ ^41^ ^,^ ^57^ ^,^ ^64^ ^,^ ^65^ ^,^ ^79^ ^,^ ^81^ ^)^. Only four studies excluded energy misreporters^(^ ^48^ ^,^ ^80^ ^)^ or energy under-reporters^(^ ^40^ ^,^ ^41^ ^)^. There was significant variation in the aspects of meal patterning examined and these aspects could be broadly categorised as: meals *v.* snacks, eating frequency, meal skipping/regularity and meal timing. These categories are used below to direct discussion on the studies' findings in relation to associations with nutrient intakes. The potential impact of different definitions on the characterisation of meal patterns and their associations with nutrient intakes is also discussed.

### Meals v. snacks in relation to nutrient intakes

A total of ten studies^(^ ^15^ ^,^ ^30^ ^,^ ^35^ ^,^ ^36^ ^,^ ^65^ ^,^ ^73^ ^,^ ^75^ ^,^ ^79^ ^,^ ^81^ ^,^ ^84^ ^)^ were identified that examined the contributions of meals and/or snacks to energy and nutrient intakes. In a prospective study of 1253 adults from the UK, Almoosawi *et al.* ^(^ ^35^ ^)^ examined 17-year changes in the contributions of breakfast, lunch and dinner to macronutrient intake. The authors found that the lunch and evening meal contributed the greatest proportion of total daily energy, protein, fat and carbohydrate intake, which was consistent over time. This is supported by other research highlighting that main meals are when the largest volume of food is normally consumed^(^ ^15^ ^,^ ^81^ ^)^. When the nutritional contribution of meals and snacks are analysed relative to their contribution to EI, a finding across five studies was that snacks provided a lower proportion of total energy from fat and/or protein than did meals^(^ ^15^ ^,^ ^36^ ^,^ ^48^ ^,^ ^65^ ^,^ ^73^ ^)^. This finding was consistent despite the difference in definitions adopted across these studies. Interestingly, two studies^(^ ^36^ ^,^ ^81^ ^)^ reported that snacks provided a greater percentage of total sugars but not total carbohydrate than meals, and Winkler *et al.* ^(^ ^81^ ^)^ noted that snacks eaten after lunchtime contained less protein and fibre than the morning snack. Two studies^(^ ^73^ ^,^ ^79^ ^)^ showed that the percentage of protein intake was highest in the evening, particularly among older adults^(^ ^73^ ^)^. This suggests that macronutrient differences between meals and snacks may be influenced by both the type and timing of food consumed.

There is a paucity of information on the relative contributions of meals or snacks to intakes of micronutrients and other dietary components. Roos & Prättälä^(^ ^65^ ^)^ examined the impact of adherence to a conventional Finnish meal pattern (breakfast, warm lunch and warm dinner plus two snacks) among 1861 adults aged 25–64 years and found, per unit of energy, that meals contributed more fibre and carotenoids but less sugar, vitamin C and alcohol than snacks. This finding remained consistent across sex and after adjustment for age and region. Additionally, a study on the adherence to a Dutch meal pattern (breakfast, morning snack, lunch bread meal, afternoon snack, hot dinner meal)^(^ ^30^ ^)^ found that the (hot) dinner meal was the main contributor to the intakes of (haem) Fe, Zn and vitamins B_1_, B_6_, B_12,_ C, D and E. Snacks may also be important in assisting populations to meet dietary guidelines for micronutrient intakes; one study^(^ ^75^ ^)^ of young adult students found that snacks contributed significantly to the percentage of the recommended daily allowances for Ca, Fe, vitamin C, thiamin, riboflavin and niacin. In the same study, snacks were important contributors of Fe and Ca intake among women, whose meal contributions of these micronutrients were only about 65 % and about 79 % of the RDA, respectively.

### Eating frequency and nutrient intakes

Of eighteen studies that examined eating frequency (including snacking frequency), fourteen found that eating frequency was associated with higher EI^(^ ^40^ ^,^ ^41^ ^,^ ^45^ ^–^ ^47^ ^,^ ^52^ ^,^ ^57^ ^,^ ^67^ ^,^ ^72^ ^,^ ^77^ ^,^ ^78^ ^,^ ^80^ ^,^ ^82^ ^,^ ^83^ ^)^. However, the evidence to support associations with nutrient intake is less consistent. Two large population-based studies, one in US adults^(^ ^52^ ^)^ and the other in Swedish adults^(^ ^80^ ^)^, found that those who ate six or more times per d had higher intakes of carbohydrate and fibre but lower intakes of fat and protein compared with adults who ate once or twice per d or less than three times per d, respectively. In these studies, a higher eating frequency was also associated with higher nutrient densities of folate, vitamin C and Fe^(^ ^52^ ^,^ ^80^ ^)^, and Ca and K^(^ ^52^ ^)^. Adjustment for multiple important sociodemographic and lifestyle-related confounders^(^ ^52^ ^)^ and exclusion of energy misreporters^(^ ^80^ ^)^ did not attenuate the significance of the results in these two studies.

Snacking frequency also appears to be an important contributor to intakes of macro- and micronutrients among older US adults aged ≥  65 years^(^ ^70^ ^,^ ^77^ ^)^. Snackers (consumers of one or more snacks per d) had significantly higher intakes of protein, carbohydrate and fat compared with non-snackers^(^ ^77^ ^)^, and a higher snacking frequency was associated with higher mean daily intakes of vitamins A, C and E, β-carotene, Mg and K^(^ ^70^ ^)^, after controlling for important confounders in both of these studies.

In contrast, three studies^(^ ^45^ ^,^ ^46^ ^,^ ^67^ ^)^ found that the proportion of energy from protein but not fat was negatively associated with snacking frequency. Additionally, Ovaskainen *et al.* ^(^ ^47^ ^)^ found that men with a snack-dominated meal pattern (defined as the majority of daily EI derived from snacks) had significantly lower fibre and micronutrient intake (vitamins A, C, E, Ca, K, Na, Fe, Mg) when compared with men with a meal-dominated pattern. The inconsistency in findings may be partly explained by how snacks have been examined relative to the overall eating pattern: that is, snack consumption in addition to main meals *v.* snacking in place of meals.

### Meal skipping and nutrient intakes

A total of six studies^(^ ^23^ ^,^ ^64^ ^,^ ^66^ ^,^ ^68^ ^,^ ^69^ ^,^ ^71^ ^)^ were identified that examined the influence of breakfast skipping on nutrient intakes and only one study^(^ ^71^ ^)^ was identified that examined the nutritional impact of omitting the lunch or dinner meal. Breakfast skipping was consistently associated with lower micronutrient intakes^(^ ^23^ ^,^ ^64^ ^,^ ^66^ ^,^ ^68^ ^,^ ^69^ ^,^ ^71^ ^)^, even after adjustment for EI and other important confounders^(^ ^23^ ^,^ ^60^ ^,^ ^66^ ^)^. Breakfast skipping was also associated with a higher prevalence of not meeting the recommended intakes for Ca^(^ ^64^ ^,^ ^66^ ^,^ ^68^ ^,^ ^69^ ^)^, vitamin C^(^ ^23^ ^,^ ^64^ ^,^ ^68^ ^,^ ^69^ ^)^, folate^(^ ^64^ ^,^ ^68^ ^,^ ^69^ ^)^, vitamin A^(^ ^23^ ^,^ ^66^ ^,^ ^68^ ^,^ ^69^ ^)^ and Mg^(^ ^23^ ^,^ ^66^ ^)^ compared with regular breakfast consumers. In addition, Williams^(^ ^69^ ^)^ found that, among older Australian adults (aged ≥  65 years), the prevalence of not meeting the recommended daily intakes for almost all nutrients examined was, among breakfast skippers, twice that of regular breakfast eaters. In a study of Japanese women students^(^ ^71^ ^)^, skipping lunch or supper was negatively correlated (*P*< 0·05) with total EI and absolute intakes of carbohydrate and vitamin K (lunch only).

### Meal timing and nutrient intakes

Only three studies were identified that examined associations between meal timing and EI^(^ ^76^ ^,^ ^79^ ^,^ ^84^ ^)^ or macronutrient intake^(^ ^76^ ^)^. In these studies, the proportion of EI consumed in the evening was positively associated with overall EI^(^ ^76^ ^,^ ^79^ ^,^ ^84^ ^)^. Among a large sample of US men and women, an increasing proportion of energy consumed after 17.00 hours was associated with an increase in mean daily alcohol intake but a decrease in mean carbohydrate intake (*P*< 0·05).

### Studies examining meal patterns and overall diet quality

A total of fourteen studies were identified that examined associations between meal patterns and measures of overall diet quality (Table 4). Most studies were conducted in the USA^(^ ^23^ ^,^ ^28^ ^,^ ^85^ ^–^ ^89^ ^)^, with fewer studies conducted in Australia^(^ ^22^ ^,^ ^56^ ^,^ ^90^ ^)^, Canada^(^ ^91^ ^,^ ^92^ ^)^, Western Europe^(^ ^93^ ^)^ and Iran^(^ ^94^ ^)^. Of the studies, seven^(^ ^22^ ^,^ ^28^ ^,^ ^56^ ^,^ ^85^ ^–^ ^88^ ^)^ used bivariate analyses to determine whether diet quality was associated with meal patterns with the purpose of identifying its role as covariate in the relationship between meal patterns and health outcomes. Meal patterns were mostly assessed using a participant-identified approach^(^ ^23^ ^,^ ^56^ ^,^ ^85^ ^,^ ^86^ ^,^ ^88^ ^–^ ^91^ ^)^; however, the methods used to measure participants' EO also varied across these studies. For example, some studies asked participants to report their EO in response to one or two questionnaire items^(^ ^85^ ^,^ ^86^ ^,^ ^88^ ^,^ ^90^ ^,^ ^91^ ^)^ whereas other studies used 24 h recall methodology^(^ ^23^ ^,^ ^89^ ^)^. Importantly, where questionnaire items were used, the reliability and/or validity of these measures were rarely reported^(^ ^56^ ^)^. Additionally, in three studies^(^ ^87^ ^,^ ^92^ ^,^ ^93^ ^)^ the approach used to define a ‘meal’ could not be readily identified.

**Table 4:** Characteristics of studies that have examined associations between meal patterns and overall diet quality

First author (year)	Country and study design	Sample	Aspect(s) of meal patterns examined	Measure(s) to assess diet and meal patterns	Meal or snack definition	Diet quality indicator(s)	Covariates	Selected key findings
Azadbakht (2013)^(^ ^94^ ^)^	Iran C/S	411 women students, 18–28 years	Breakfast skipping	FFQ Meal patterns: not described	Time of-day*	HEI, DDS	Not clear if covariates were adjusted for in the multivariate ANOVA	HEI and DDS scores and diversity scores for fruits, vegetables and whole grains were significantly lower among breakfast skippers than consumers (*P*< 0·001)
Deshmukh-Taskar (2010)^(^ ^23^ ^)^	USA C/S	2615 men and women, 20–39 years	Breakfast skipping	1 × 24HR	Participant-identified*	HEI	Ethnicity, sex, sex × ethnicity, age, poverty income ratio, smoking status, marital status and PA	Breakfast skippers had significantly lower (*P*< 0·0001) HEI scores than those who consumed ready-to-eat breakfast cereals or other breakfast foods
Dewolfe (2003)^(^ ^91^ ^)^	Canada C/S	84 men and 21 women, ≥ 65 years	Meal skipping and snacking	3 × 24HR Meal patterns: Q	Participant-identified	Diet score based on compliance with national dietary guidelines	Preparing own meals, how well food tastes, prescription medication use, sex	Eating lunch daily was positively associated (standardised β = 0·24, 95 % CI 0·05, 0·42) with the diet score reflecting adherence to Canadian dietary guidelines
Cahill (2013)^(^ ^28^ ^)^	USA Prospective (16-year follow-up)	29 209 health professional men, 40–75 years	Breakfast eating and late-night eating	FFQ† Meal patterns: Q	Time-of-day	AHEI-2010	–	Based on age-standardised baseline data, no significant differences in AHEI scores were reported between breakfast consumers and non-breakfast consumers or late-night eaters and non-late-night eaters
Kim (2011)^(^ ^88^ ^)^	USA/Puerto Rico C/S	27 983 women, 35–74 years	Snack dominance and conventional eating pattern	Modified block FFQ† Meal patterns: Q	Participant-identified*	HEI	–	A higher conventional eating score (eating meals and snacks during conventional times) was associated with higher HEI scores (*P*< 0·01) whereas a higher snack-dominant eating score was associated with lower HEI scores (*P*< 0·01)
Mekary (2012)^(^ ^85^ ^)^	USA Prospective (14-year follow-up)	34 968 men, 40–75 years	Eating frequency	FFQ† Meal patterns: Q	Participant-identified	DASH score	–	Based on age-standardised baseline data, there was a positive association between eating frequency and the DASH score (*r* 0·14)
Mekary (2013)^(^ ^86^ ^)^	USA Prospective (6-year follow-up)	46 289 women	Eating breakfast regularly and eating frequency	FFQ† Meal patterns: Q (two items)	Participant-identified*	AHEI-2010	–	Based on age-standardised baseline data, women who ate breakfast ≤ 6 times/week had lower scores for the AHEI-2010 than regular breakfast consumers. Diet quality by eating frequency was not assessed
Mesas (2012)^(^ ^93^ ^)^	Spain C/S	10 791 men and women, ≥ 18 years	Skipping breakfast	Diet history Q† Meal patterns: Q	Never eating anything at the breakfast occasion (meal definition could not be established)	MEDAS score; the OmniHeart diet score	Age, sex, education, social class, smoking, alcohol, binge drinking, PA at work, BMI and morbidity	No significant associations were found between skipping breakfast and either the MEDAS score or the OmniHeart diet score
Odegaard (2013)^(^ ^87^ ^)^	USA Prospective (follow-up: 18 years)	3598 men and women, 18–30 years at baseline	Breakfast frequency	Diet history Q† Meal patterns: Q	No definition provided	*A priori* diet quality score (no specific name given)	–	Based on C/S data at the 7-year follow-up, higher levels of breakfast intakes were associated with higher diet quality scores
Shatenstein (2013)^(^ ^92^ ^)^	Canada C/S	853 men and 940 women, 67–84 years	Meal frequency (snacks not included)	3 × 24HR Meal patterns: Q	No definition provided	Canadian HEI	Sex-specific models. Inclusion of the following covariates depended on model: education, diet, income, alcohol, wears dentures, perceived physical health, eats in restaurants, nutrition knowledge, hunger, BMI, chewing problems	Among males and females, number of meals/d was positively associated with Canadian HEI scores (β = 1·91, *P*< 0·02 and β = 3·61, *P*< 0·0001, respectively)
Smith (2010)^(^ ^22^ ^)^	Australia Prospective	1020 men and 1164 women, 9–15 years at baseline and 26–36 years at follow-up	Breakfast skipping	FFQ Meal patterns: Q (meal patterns chart)	Participant-identified and time-of-day	Compliance with dietary advice in the Australian Guide to Healthy Eating	–	Participants who skipped breakfast in both childhood and adulthood were less likely to meet recommendations for fruit, dairy products, lean meat and alternatives and takeout foods (*P*< 0·001) than those who did not skip breakfast at either time point
Smith (2012)^(^ ^56^ ^)^	Australia C/S	1273 men and 1502 women, 26–36 years	Eating frequency	FFQ Meal patterns: Q (meal patterns chart)	Participant-identified	Diet score based on compliance with national dietary guidelines	Stratified by sex	There was a positive association (*P*< 0·001) between daily eating frequency and dietary scores, and meeting recommendations for fruit and dairy products among both men and women
Smith (2013)^(^ ^90^ ^)^	Australia C/S	4123 women from low-SES areas, 18–45 years	Breakfast skipping	FFQ Meal patterns: Q (one item)	Participant-identified	DGI	–	Compared with women who ate breakfast < 1 d/week or 1–2 d/week, those who ate breakfast ≥ 3 d/week were more likely to be in the highest tertile for DGI scores
Zizza (2012)^(^ ^89^ ^)^	USA C/S	11 209 adults ≥ 20 years	Snack frequency	1 × 24HR	Participant-identified*	HEI-2005	Sex, race or ethnicity, education, smoking status, PA, eating ≥ 3 meals/d, chronic diseases, age, BMI, energy from meals	Frequency of snacking was positively associated with HEI-2005 scores and intakes of whole fruit, whole grains, milk, oils and Na (all *P*< 0·001) but inversely associated with total vegetables (*P*= 0·009), solid fat and added sugars (*P*= 0·007)

C/S, cross-sectional; HEI, Healthy Eating Index; DDS, dietary diversity score; 24HR, 24 h recall; PA, physical activity; Q, questionnaire; AHEI, Alternative Healthy Eating Index; DASH, Dietary Approaches to Stop Hypertension; MEDAS, Mediterranean Diet Adherence Score; OmniHeart, Optimal Macronutrient Intake Trial to Prevent Heart Disease; SES, socio-economic status; DGI, dietary guidelines index.

* Beverages could explicitly qualify as a separate eating occasion.

† Excluded individuals with implausible energy intakes.

The most common measure used to assess overall diet quality was a previously validated and reliability tested *a priori* diet quality index which reflects an individual's adherence to the dietary guidelines for the country of the sample population (for example, the Healthy Eating Index (HEI), the Alternative HEI (AHEI) and the Dietary Guidelines Index (DGI))^(^ ^23^ ^,^ ^28^ ^,^ ^86^ ^,^ ^88^ ^–^ ^90^ ^,^ ^92^ ^,^ ^94^ ^)^. The measures used in the remaining studies were varied and included scores that measured adherence to: a traditional Mediterranean diet (MEDAS score)^(^ ^93^ ^)^; Dietary Approaches to Stop Hypertension (DASH) diet score^(^ ^28^ ^)^; a dietary approaches to prevent heart disease diet score (Optimal Macronutrient Intake Trial to Prevent Heart Disease (OmniHeart) score)^(^ ^5^ ^)^; hypothesised healthy eating patterns (*a priori* diet score)^(^ ^87^ ^)^; and national guidelines for healthy eating^(^ ^22^ ^,^ ^56^ ^,^ ^91^ ^)^. The associations between these diet quality measures and different meal patterns are discussed below.

### Eating frequency and diet quality

Few studies have examined associations between eating frequency (including meal and/or snack frequency) and diet quality. Among US male health professionals, a higher eating frequency was associated with higher DASH scores, reflecting higher diet quality (*r* 0·14; no *P* value provided)^(^ ^85^ ^)^. A higher meal frequency was also associated with higher diet quality as measured by the Canadian HEI among older male and female adults aged 67–84 years old (men: β 1·91, *P*< 0·02; women: β 3·61, *P*< 0·0001)^(^ ^91^ ^)^. Of note, neither of these studies adjusted for total EI. The mean score for HEI-2005 also increased with increased daily snacking frequency (for example, no snacks = 49·3 (se 0·5) *v.* ≥  4 snacks = 51·5 (se 0·6), *P*< 0·001) among US adults, after adjustment for sociodemographic factors, BMI, eating three or more meals daily and EI from meals^(^ ^89^ ^)^. Conversely, another study reported no association between snacking between meals and diet quality^(^ ^91^ ^)^, and Kim & Kim^(^ ^83^ ^)^ found that a HEI score significantly decreased according to each increased quartile of a snack-dominant eating score (high snack frequency and low meal frequency) (*P*< 0·01). Again, neither study reported adjustment for total EI.

### Meal skipping/regularity and diet quality

Of the nine studies identified that examined associations between skipping breakfast and diet quality, six found a negative association^(^ ^22^ ^,^ ^23^ ^,^ ^86^ ^,^ ^87^ ^,^ ^90^ ^,^ ^94^ ^)^ and three found no association^(^ ^28^ ^,^ ^91^ ^,^ ^93^ ^)^. However, the lack of association in two of these latter studies may be explained by their respective study populations; overall diet quality was high among men in the US Health Professionals Follow-up study^(^ ^28^ ^)^ while Dewolfe & Millan^(^ ^91^ ^)^ used a small convenience sample of eighty-four female and twenty-one male older adults from a single region in Canada. In the latter study^(^ ^91^ ^)^, eating lunch daily was associated with higher diet quality scores that assessed compliance with the Canadian Guide to Healthy Eating. No other studies were identified that have examined skipping/regularity of meals other than breakfast.

### Meal timing and diet quality

Studies examining associations between meal timing and diet quality are rare. In the US Health Professionals Follow-up study, Cahill *et al.* ^(^ ^28^ ^)^ found no association between late night eating (defined as eating after going to bed) and AHEI scores; however, as mentioned previously, the authors acknowledged that AHEI scores were high in this sample, irrespective of their reported meal patterns.

### Potential impact of different meal definitions on the characterisation of meal patterns and associations with nutrient intakes and diet quality

Clear and objective definitions of what is a meal and what is a snack are critical for determining the energy and nutrient contributions of meals *v.* snacks, meal skipping or meal timing. Without a clear definition misclassification bias is likely, thus affecting the interpretation of associations with nutrients both within and across studies. In allowing participants to identify meals and snacks, subjective decision-making is inherently present. Previous research suggests that situational cues such as the type, quality or amount of food and the presence of others may affect a participant's decision to classify an EO as a meal or a snack^(^ ^95^ ^)^. It is also unclear whether the same meal or snack situation would be classified similarly by different individuals; research in this area is needed in order to better understand the between-subject variation when applying a participant-identified definition. While a time-of-day approach can be applied consistently for all participants, it may not capture meals and snacks eaten at varied times. Furthermore, it is unknown whether meals and snacks would be classified similarly if either a participant-identified or a time-of-day approach were applied. Research on the comparability of the different definitions that seek to define meals and snacks would help address this issue.

It is important to note that studies that have examined eating frequency (including meal and/or snack frequency) differ in both the methods used to define meals and snacks and the time-gap to separate individual EO, which may make an impact on the frequency of the respective EO reported. For example, while most meal or snack definitions include beverages alongside food, not all studies explicitly considered a beverage-only occasion as a separate EO^(^ ^40^ ^,^ ^41^ ^,^ ^67^ ^,^ ^72^ ^,^ ^78^ ^,^ ^82^ ^)^. In addition, larger time intervals used to separate EO may result in EO, including beverage-only occasions, being overlooked and this may affect associations between eating frequency and energy and nutrient intakes. For example, Kant *et al.* ^(^ ^96^ ^)^ demonstrated a positive association between 24 h beverage EI with saturated fat, sugar, Na and alcohol intakes, after adjustment for EI from foods. However, a definition that excludes ‘low-energy’ beverage-only EO (for example, < 210 kJ) may also be important. A recent study^(^ ^55^ ^)^ showed that, compared with a definition that included all energy-containing EO, there was a stronger correlation between eating frequency and EI after applying a definition that used a minimum energy criterion of ≥  210 kJ (men: *r* 0·45 *v.* 0·53; women *r* 0·39 *v.* 0·57, respectively), which remained after excluding energy misreporters.

As few studies have examined associations between meal patterns and diet quality, the impact of different meal definitions is difficult to assess. Breakfast skipping was consistently inversely associated with diet quality in six out of nine studies, despite the different definitions used (time-of-day^(^ ^88^ ^,^ ^94^ ^)^ and participant identified^(^ ^22^ ^,^ ^23^ ^,^ ^28^ ^,^ ^86^ ^,^ ^90^ ^)^), and in some cases, no clear definition was provided^(^ ^87^ ^,^ ^93^ ^)^.

Of note, many studies that have examined meal patterns and diet quality also used questionnaire items with unreported reliably and validity to collect meal pattern data. The lexical and semantic features of questionnaire items can differ between studies and may influence participant responses^(^ ^97^ ^)^. For example, items may ask participants to indicate the times of the day they usually eat^(^ ^28^ ^)^ or how many days they usually have something to eat for breakfast^(^ ^90^ ^)^, whereas other items provide additional instruction such as include all beverages^(^ ^88^ ^)^ or all nutritive beverages^(^ ^86^ ^)^. Questions that use the word ‘eat’ but provide no additional examples or cues as to what to include may therefore only elicit information about food-only EO or combined food and beverage EO but not beverage-only EO. However, until questionnaire items are validated against a pre-existing valid method (for example, a 24 h recall), how accurately they capture meal, snack and all EO (including beverages) remains unclear.

## Discussion

Meal patterns are multidimensional and can be described according to their patterning, format and context. However, due to the limited dietary assessment methods available, most research has focused on meal patterning. To date, a variety of definitions has been used to examine meal patterns. In addition, a number of additional criteria have been adopted in meal pattern research, which may have an impact on the types of meal patterns reported and described in the literature. Although over the past few decades there has been general consensus that a universally accepted definition of a meal is crucial^(^ ^54^ ^,^ ^98^ ^)^, there have been few attempts to define meals in a consistent and standardised way.

Research suggests that different meal and/or snack patterns are related to both nutrient intakes and overall diet quality, with the most consistent finding being an inverse relationship between skipping breakfast and nutrient intakes/diet quality. Skipping meals other than breakfast has rarely been examined but may be important, particularly for vulnerable groups such as the elderly^(^ ^69^ ^,^ ^91^ ^)^. In addition the nutritional impact of snack, meal and overall eating frequency remains unclear and little research has looked at the how meal timing influences nutritional intake/overall diet quality. This may be an important area of research in light of preliminary evidence suggesting that the timing of energy and/or macronutrient intake during the day is associated with cardiometabolic risk^(^ ^27^ ^,^ ^28^ ^,^ ^99^ ^,^ ^100^ ^)^.

The conflicting findings for the associations between eating frequency (including snack/meal frequency) and nutrient intake/diet quality may be, in part, attributed to not only the heterogeneity of meal patterns examined, but also to different definitions of meals and snacks. While meals and snacks are hypothesised to exert different effects on EI and nutrient intake, some researchers suggest that the sociocultural and value-laden nature of the terms used to identify different meals and snacks precludes such delineation^(^ ^101^ ^)^. Although it is widely acknowledged that different definitions used to define meals/snacks are likely to hamper interpretation of findings across studies^(^ ^8^ ^,^ ^44^ ^)^, research explicitly examining the impact of these different definitions is rare^(^ ^55^ ^)^. There has been little attempt to examine meal patterns in a consistent and standardised way.

Another important consideration for future research examining eating frequency is potential overlap in the meal patterns that are being examined, which further complicates comparisons between studies. For example, a study that examines eating frequency comparing eating one or two times per d *v.* four to six times per d is also encapsulating meal skipping and meal patterns with snacks, respectively. That is, depending on cultural norms, an individual who only eats one or two times per d may also be considered to be skipping one or two EO. Categorising individuals as being high snack consumers may include individuals who consume snacks in lieu of meals, and therefore future research should consider eating frequency and/or snack frequency in the context of meal frequency/skipping to better differentiate the impact of different types of meal patterns. Some evidence^(^ ^80^ ^)^ also suggests that healthy and unhealthy dietary patterns can exist among individuals who are high-frequency snack consumers. This may also partially explain the lack of consistent findings for the association between snack frequency and nutrient intakes, and therefore future research should consider examining meal patterns in the context of a individual's overall dietary pattern. Measures of eating frequency may include beverage-only occasions, however, these types of EO have not always been considered when examining the relationship between meal patterns and nutritional intake. This may be an important consideration given the emerging evidence of sugar-sweetened beverages (SSB) in the aetiology of obesity^(^ ^102^ ^,^ ^103^ ^)^ and cardiometabolic risk^(^ ^104^ ^,^ ^105^ ^)^. Moreover, beverage-only occasions may be especially relevant among certain subgroups of the population; for example, adolescent and young adult males have been shown to be high consumers of SSB^(^ ^106^ ^)^.

A limitation of the literature to date on diet quality is that the primary purpose of many of the included studies was to examine associations between meal patterns and health outcomes. Therefore, few of these studies adjusted associations for total EI and important sociodemographic and lifestyle factors. Another limitation of these studies was that meal patterns were often assessed using simple questionnaires with unreported reliability or validity. Importantly, questions regarding meals in questionnaires may not be well defined and this may extend to how respondents should consider beverages.

Under-reporting of EI is a common and well-known limitation of studies that assess dietary intakes^(^ ^107^ ^)^. Despite this, very few studies on meal patterns have examined the impact of energy misreporting. As eating frequency is positively related to EI, it may be that those who under-report EI also under-report their eating frequency^(^ ^45^ ^,^ ^108^ ^)^. There is also some evidence that snacks are more prone to being under-reported^(^ ^108^ ^)^. Results from a pooled analysis of five large validation studies showed that under-reporting of EI with a single 24 h recall was approximately 15 %^(^ ^109^ ^)^. Unless adjusted for, energy misreporting may obscure important relationships between meal patterns, nutrient intakes/diet quality and, ultimately, health outcomes^(^ ^48^ ^)^.

### Recommendations to advance the field

To advance the area of meal pattern research, the methods used to collect meal pattern data require further development. Measures that are inexpensive to administer and have low participant burden (for example, questionnaire items) need to be developed and tested for reliability and validity. Contextual information is not always collected as part of a 24 h recall, yet additional questions about eating location and activities while eating^(^ ^110^ ^)^ could be considered in order to better understand the contextual factors that influence associations between meal patterns and diet quality. While specific food records have been adapted to collect contextual information (for example, the Weekly Food Diary method^(^ ^58^ ^)^), this method also involves a high participant burden. Dietary assessment methods that utilise new technology (for example, smartphones) may assist in the development of meal pattern research. Devices that people use and carry alongside them every day with the added capacity of a personal digital assistant used in a previous study^(^ ^32^ ^)^ may be a low burden and efficient way to collect meal pattern data in ‘real time’. A major advantage of such technology would be that researchers could collect information allowing examination of all three meal pattern constructs: patterning, format and context. Furthermore, rich contextual data collected in real time could provide insight into the factors that influence participants' decisions to classify an EO as a meal or snack and therefore help in refining existing meal definitions. Currently little research has examined meal format; however, understanding how different combinations of foods in a meal influence overall diet quality could be an important step in developing a meals-based framework for dietary guidelines. Further work is also required in developing and applying innovative statistical techniques to examining meal patterns, with few applications tested in the literature.

However, it is important to acknowledge that developing new methods to collect and analyse meal patterns data will take considerable time. A major issue still remains of the different definitions available to researchers when conducting meal patterns research. Further analysis (for example, sensitivity analysis) that examines more than one definition from the current literature would facilitate understanding of how the choice of definition makes an impact on the characterisation of meal patterns and associations with outcomes such as nutrient intake and diet quality.

## Conclusion

Overall, there are a number of gaps and limitations in meal pattern research that need to be addressed to further our understanding of how meal definitions influence the characterisation of meal patterns, and the contribution of different meal patterns to nutrient intake and overall diet quality. While current evidence suggests breakfast skipping may be detrimental to diet quality, the nutritional impact of eating frequency, skipping meals other than breakfast and meal timing is inconclusive and warrants further investigation. Future studies should consider how different contexts, beverage-only occasions and energy misreporting affect the relationship between meal patterns and diet quality. The heterogeneity of meal definitions is a major impediment to the interpretation of findings across studies in this field of research. Future research that examines the influence of different meal definitions on the characterisation of meal patterns will facilitate the interpretation of the existing literature, and provide recommendations on the most appropriate methods to advance the field.
